# Hearing preservation in children with electric-acoustic stimulation after cochlear implantation: Outcome after electrode insertion with minimal insertion trauma

**DOI:** 10.1007/s00106-018-0532-3

**Published:** 2018-08-21

**Authors:** T. Rader, A. Bohnert, C. Matthias, D. Koutsimpelas, M-A. Kainz, S. Strieth

**Affiliations:** 1 Audiological Acoustics Division, Department of Otolaryngology, Head and Neck Surgery, University of Mainz, Langenbeckstraße 1, 55131 Mainz, Germany; 2 Department of Otolaryngology, Head and Neck Surgery, University of Mainz, Langenbeckstraße 1, 55131 Mainz, Germany

**Keywords:** Hearing loss, Cochlear implantation, Ear, inner, Audiometry, Pure-tone, Child

## Abstract

**Background:**

Cochlear implantation in patients with functional residual low-frequency hearing is performed according to an established hearing-preserving surgical technique in order to cause minimal trauma of inner ear structures. Due to the increasing number of cochlear implants in children, the preservation of residual hearing is becoming increasingly important in this patient collective.

**Objectives:**

Short- and mid-term hearing preservation outcome in pediatric patients is investigated.

**Materials and methods:**

A group of 9 children (12 ears) between 5 and 12 years of age were examined after hearing-assisted cochlear implantation with respect to the pure tone audiometric thresholds. Retrospectively, short-term hearing preservation (up to 3 months after surgery) was examined. In a subgroup of 5 children, mid-term hearing preservation (7.5 to 16 months after surgery) was also analyzed. The mean values of hearing preserved (HL%) and hearing loss (HL) due to electrode insertion were calculated as measured values.

**Results:**

In the whole group, the mean values of the preoperative PTA_low_ were 29.8 dB and the short-term postoperative PTA_low_ 42.6 dB. The mean value of the HL% was 73.6%, corresponding to an HL of 9.4 dB. In the subgroup, the mean PTA_low_ postoperatively was 46.0 dB in the mid-term and the HL% at 80.7% with a HL of 6.6 dB.

**Conclusions:**

The results in children are consistent with the results in adults. Electric-acoustic stimulation (EAS) should be used in the treatment of children with existing low-frequency residual hearing, as good residual hearing preservation can also be achieved in children after implantation.

## Background

If low-frequency residual hearing in subjects with severe high-frequency hearing loss (HL) cannot successfully be treated with hearing aids, electric-acoustic stimulation (EAS) can be used in the same ear, creating a synergistic effect.

This has a positive impact on speech understanding [8–10, 15]. Compared to bilateral cochlear implants (CI) with only electric stimulation, EAS users show considerably better speech understanding [6, 11, 16, 17]. Due to the development of structure-preserving electrode arrays and surgical procedures based on the hearing preservation cochlear implantation (HPCI) standard [1, 24, 27, 29], residual hearing can be preserved postimplantation in adults with only minor losses. Mechanical and acoustic trauma to the fine structures in the cochlea are minimized (soft surgery) and acoustic hearing in the low frequencies can be preserved in the medium term after cochlear implantation in most patients [8, 13]. Preliminary studies have also shown the possibility of successful hearing preservation in children [4, 5, 12].

Due to the constantly improving clinical diagnosis of hearing disorders in children, frequency-specific hearing thresholds can be assessed with precision in an increasing number of children. Thus, the number of hearing preservation cochlear implantations is steadily increasing in children and residual hearing is gaining in importance in this patient population. Considering the long life expectancy of this patient population and the first experimental achievements in the development of new approaches for the improvement of the electrode/auditory nerve interface [23] or the new approaches in hair cell regeneration [21], structure preservation of the cochlea is essential.

The international HEARRING Group expert group recommends applying the HPCI standard in all cochlear implantations in children, regardless of the extent of residual hearing [20].

## Materials and methods

### Patient population

All children with low-frequency residual hearing implanted with electrode arrays specifically designed for hearing preservation at the Medical University Clinic of Mainz between 2012 and 2016 were included in the retrospective data analysis. Pure tone audiometry was performed in a group of 9 children (12 ears) aged between 5 and 12 years after hearing preservation cochlear implantation (Table 1).

**Table 1:** Demographic data of the 9 children (12 ears) with hearing preservation cochlear implantation

Ear ID	Patient no.	Implant side	Age at implantation (years)	Sex	Brand	Implant	Electrode	Etiology
E01	01	Left	6	Male	MED-EL	CONCERTO	Flex24	Ototoxic
E02	02	Left	7	Male	MED-EL	CONCERTO	Flex24	Unknown
E03	03	Right	5	Female	MED-EL	CONCERTO	Flex24	Congenital
E04	03	Left	6	Female	MED-EL	CONCERTO	Flex24	Congenital
E05	04	Right	10	Female	MED-EL	SYNCHRONY	Flex24	Progressive
E06	05	Right	5	Female	MED-EL	SYNCHRONY	Flex24	Progressive
E07	06	Left	5	Female	MED-EL	SYNCHRONY	Flex24	Progressive
E08	07	Right	12	Male	MED-EL	SYNCHRONY	Flex24	Progressive
E09	05	Left	6	Female	MED-EL	SYNCHRONY	Flex24	Progressive
E10	08	Left	9	Female	MED-EL	SYNCHRONY	Flex24	Ototoxic
E11	01	Right	11	Male	MED-EL	SYNCHRONY	Flex24	Ototoxic
E12	09	Left	12	Male	Cochlear	CI522	Slim Straight	Progressive

*ID* identification number, *Med-EL* MED-EL, Innsbruck, Austria, *Cochlear* Cochlear, Macquarie University, NSW, Australia

Mean age at implantation was 8.3 years; 4 were masculine. Three children were bilaterally provided with EAS, 4 only in the left ear and 2 only in the right ear. Etiology of the HL was unknown in 7 of 12 ears; the HL was progressive in the high-frequencies in 5 ears. The HL was caused by ototoxicity in 3 ears, while the HL was probably congenital in 2 ears. Eleven ears were implanted with a CI and a Flex24 electrode array (MED-EL, Innsbruck, Austria). The Flex24 electrode array features 12 platinum contacts (5 apical one-sided, 7 basal double-sided) over a total length of 20.9 mm with an electrode spacing of 1.9 mm. The insertion depth is 24 mm with an electrode diameter of 0.36–0.48 mm at the apical end and 0.8 mm at the basal end.

One ear was implanted with a CI522 implant (COCHLEAR, Macquarie University, NSW, Australia) with a Slim Straight electrode array. The Slim Straight electrode array features 22 one-sided platinum contacts on a total active spacing of 20 mm with maximum full insertion of 25 mm. The electrode diameter is 0.3 mm at the apical end and 0.6 mm at the basal end.

The decision on the type of implant system was made after neutral counselling to the parents. The decision on the type of electrode array was made based on an interdisciplinary agreement within the CI team of the ENT clinic, taking into account the anatomy of the cochlea using computed tomography (CT) and magnetic resonance imaging (MRI) and the audiogram.

All implanted electrode arrays are suitable and approved for hearing preservation surgery [7, 8, 19]. The preoperative audiograms were within the EAS indication range of the corresponding CI electrode array of the respective specifications of the implant manufacturer. In all children, the best possible provision with hearing aids was evaluated before implantation according to the consensus paper of the German Society of Phoniatrics and Pediatric Audiology (Deutsche Gesellschaft für Phoniatrie und Pädaudiologie) [28]. All implantations were performed according to the HPCI standard with insertion of the electrode array through the round window.

### Audiological data analysis

First, the short-term hearing preservation (“postoperative short-term”) was analyzed between 2 weeks and 3 months (mean: 6 weeks) postimplantation to detect potential hearing defects caused during implantation. In a subgroup of 5 children, data for the evaluation of the medium-term hearing preservation (“postoperative medium-term”) were available for the period between 7.5 and 16 months (mean: 12.6 months) postimplantation to detect potential progressive hearing loss. For all 12 ears, the preoperative PTA_low_ (pure tone average for low frequency) was calculated as a mean value of the audiometric thresholds at 0.125, 0.250, and 0.5 kHz at the different times of observation. Additionally, hearing preservation was calculated in percent (HL%) following Skarzynski et al. [25] (Eq. 1) and the additional HL caused by the insertion of the electrode array across the entire frequency range (125–8000 Hz) at the different times of observation: “postoperative short-term” (Eq. 2) and “postoperative medium-term” (Eq. 3). The PTA_mdh_ variable describes the maximum output level of the audiometer for the measured audiometric frequencies.

1 $$\boldsymbol{HL\% }=[\mathrm{1}-((\mathrm{PTA}_{\text{postoperative}}-\mathrm{PTA}_{\text{preoperative}})/(\mathrm{PTA}_{\mathrm{mdh}}-\mathrm{PTA}_{\text{preoperative}}))]\times \mathrm{100}[\text{\% }]$$

2 $$HL_{\mathrm{1}}=\mathrm{PTA}_{\text{total postoperative short-term}} - \mathrm{PTA}_\text{total preoperative}$$

3 $$HL_{\mathrm{2}}=\mathrm{PTA}_{\text{total postoperative medium-term}}-\mathrm{PTA}_{\text{total preoperative}}$$

### Statistical analysis

The variables of the total group were tested for normal distribution using the Kolmogorov–Smirnov test for the “preoperative” and the “postoperative short-term” times of observation and analyzed for significant differences using the paired sample t‑test. Due to the small number of cases, the data of the subgroup for the “postoperative medium-term” follow-up were not included in the statistical analysis for significant differences. The statistical analysis was performed using the SPSS statistics 23 software (IBM, Armonk, NY, USA). *P*-values < 0.05 were considered statistically significant.

## Results

### Audiometric data

**Fig. 1:**
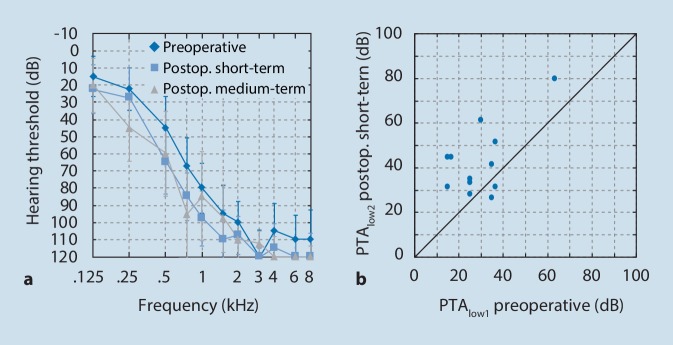
**a** Presentation of the averaged audiometric thresholds (median and interquartile range) for the different times of observation “preoperative” (*dark blue*, *n* = 12), “postoperative short-term” (*light blue*, *n* = 12), and “postoperative medium-term” (*gray*, *n* = 5). **b** Change in the preoperative averaged low-frequency thresholds PTA_low_ after cochlear implantation at the “postoperative short-term” follow-up (*n* = 12)

Audiometric thresholds of 9 children (12 ears) aged between 5 and 12 years were measured before and after hearing preservation cochlear implantation. The averaged audiometric thresholds of the three different times of observation, “preoperative”, “postoperative short-term”, and “postoperative medium-term”, are presented in Fig. 1a, including median and interquartile range.

### Short-term hearing preservation

The PTA_low_ averaged low-frequency thresholds are presented in Table 2 and significantly (T = −3.408; df = 11; *p* = 0.006) deteriorated by 12.8 dB between “preoperative” (PTA_low1_ 29.8 dB) and “postoperative short-term” (PTA_low2_ 42.6 dB) due to the surgical insertion of the CI electrode array. The measured values varied between the subjects from a deterioration of 31.6 dB (E07) to an improvement of 8.4 dB (E01). The short-term change in the averaged low-frequency thresholds PTA_low1_ measured postoperatively is presented in Fig. 1b.

**Table 2:** Individual results (*n* = 12) and average values at two time points

Ear ID	Pure tone average for low frequency		Hearing preservation	Hearing loss
	PTA_low1_ (dB)	PTA_low2_ (dB)	HL%_1_ (%)	HL_1_ (dB)
E01	35.0	26.6	104.0	−2.3
E02	63.3	80.0	71.5	6.8
E03	36.6	51.6	70.4	13.9
E04	25.0	28.3	90.2	3.0
E05	35.0	41.6	66.7	10.5
E06	36.6	31.6	102.0	−0.7
E07	30.0	61.6	39.8	22.7
E08	25.0	33.3	93.2	3.0
E09	15.0	31.6	65.1	13.2
E10	16.6	45.0	61.4	11.6
E11	15.0	45.0	50.7	16.4
E12	25.0	35.0	68.1	14.8
Mean	29.8	42.6	73.6	9.4

*ID* identification, *PTA*_*low1*_ pure tone average for low frequency, “preoperative”, *PTA*_*low2*_ pure tone average for low frequency, “postoperative short-term”, *HL%*_*1*_ percent hearing preservation, “preoperative”, *HL*_*1*_ hearing loss, “postoperative short-term”

The mean percentage of hearing preservation in the low frequencies based on Eq. 1 at the “postoperative short-term” follow-up (HL%_1_) was 73.6% (variation: 39.8 to 104%). This corresponds to a mean HL_1_ of 9.4 dB across all frequencies (range: −2.3 to 22.7 dB).

### Medium-term hearing preservation

Medium-term hearing preservation results were available for a subgroup of 5 children with a mean age of 7.3 years (E01–E05). Analyzing the results of the subgroup over time, the averaged threshold deteriorated in the low frequencies between the preoperative measurement with 39.0 dB and the postoperative short-term follow-up with 45.6 dB and the postoperative medium-term follow-up with 46.0 dB. The most distinct deterioration (6.7 dB) of the auditory threshold was measured at the postoperative short-term follow-up. In the medium term, the auditory threshold only deteriorated by 0.4 dB (Table 3).

**Table 3:** Individual results (*n* = 5) and mean values at three time points

Ear ID	Pure tone average for low frequency			Hearing preservation	Hearing loss	Hearing preservation	Hearing loss
	PTA_low1_ (dB)	PTA_low2_ (dB)	PTA_low3_ (dB)	HL%_1_ (%)	HL_1_ (dB)	HL%_2_ (%)	HL_2_ (dB)
E01	35.0	26.6	25.0	104.0	−2.3	91.8	2.7
E02	63.3	80.0	73.3	71.5	6.8	92.7	4.5
E03	36.6	51.6	40.0	70.4	13.9	98.0	0.9
E04	25.0	28.3	30.0	90.2	3.0	93.4	2.0
E05	35.0	41.6	61.6	66.7	10.5	27.5	22.7
Mean	39.0	45.6	46.0	74.7	6.4	80.7	6.6

*ID* identification, *PTA*_*low1*_ pure tone average for low frequency, “preoperative”, *PTA*_*low2*_ pure tone average for low frequency, “postoperative short-term”, *PTA*_*low2*_ pure tone average for low frequency, “postoperative medium-term”, *HL%*_*1*_ percent hearing preservation, “preoperative”, *HL*_*1*_ hearing loss, “postoperative short-term”, *HL%*_*2*_ percent hearing preservation, “postoperative medium-term”, *HL*_*2*_ hearing loss, “postoperative medium-term”

The mean percentage of hearing preservation HL%_1_ was 74.7% at the postoperative short-term follow-up (range: 66.7–104.0%) and 80.7% at the postoperative medium-term follow-up (range: 27.5–98.0%). This results in a mean HL_1_ of 6.4 dB (range: −2.3 to 13.9 dB) across all frequencies at the postoperative short-term follow-up and of 6.6 dB (range: 0.9–22.7 dB) across all frequencies at the postoperative medium-term follow-up.

**Fig. 2:**
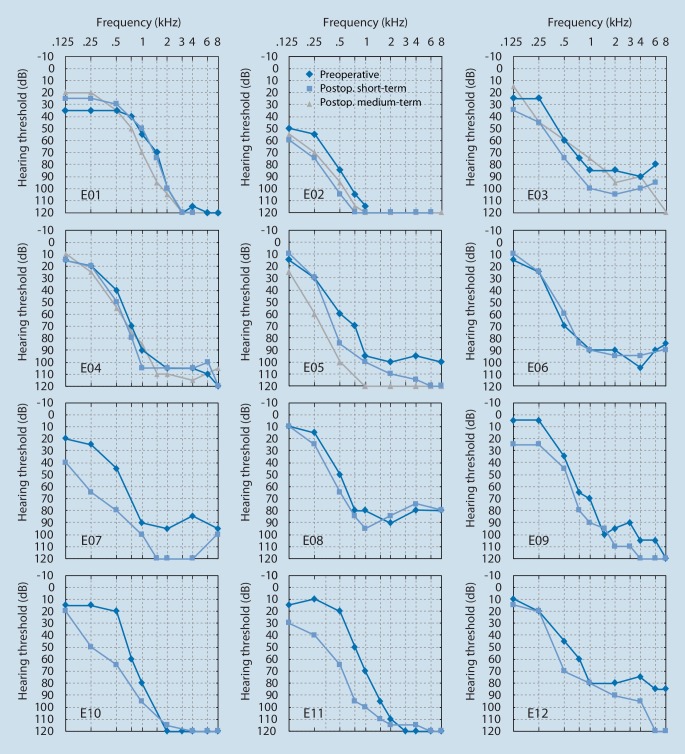
Individual presentation of the audiometric results of all ears (*n* = 12; *E01* to *E12*) at the different times of observation: “preoperative” (*dark blue*), “postoperative short-term” (*light blue*), and “postoperative medium-term” (*gray*)

The individual audiograms of all subjects are presented in Fig. 2 for all times of observation (preoperative, postoperative short-term, postoperative medium-term).

## Discussion

The hearing preservation cochlear implantation has become an established procedure in the treatment of patients with severe to profound hearing loss with usable low-frequency residual hearing. Before implantation, it should be verified if the best possible state-of-the-art hearing aid provision was offered [28].

This study showed that a high percentage of hearing preservation can be achieved in children after cochlear implantation following the HPCI standard in 73.6% of all cases and in up to 80.7% of all cases in the analyzed subgroup.

### Hearing preservation in adults

Many studies on adult patients have shown that low-frequency hearing preservation can be achieved and that EAS is an option in cochlear implantation in the medium and long term. Helbig et al. [8], for example, showed in a study with 96 patients (103 ears) postoperative “complete hearing preservation” (definition: deterioration of PTA_low_ ≤ 10 dB) in 31.1%, “partial hearing preservation” (definition: deterioration of 10–30 dB) in 47.6%, and “minimum hearing preservation” (definition: deterioration of PTA_low_ ≥ 30 dB) in 13.6%. Complete HL was detected in 7.8% of all patients. No correlations between the hearing results and etiology, design of the electrode array or surgical approach were identified.

Compared to the data presented in this study, the study of Mertens et al. [13] showed similar hearing preservation results in 9 patients (11 ears). The average percentage of hearing preservation of 48% was slightly lower, but the observation period of between 6 months and 10 years postimplantation was longer.

Roland et al. showed no significant deterioration of the postoperative low-frequency thresholds (PTA_low_) after 5 years of observation in 32 ears and provision with Cochlear Nucleus Hybrid L24 electrodes [22]. It should be mentioned, however, that the insertion depth of the L24 electrode arrays with a length of 16 mm is considerably less than with the electrode arrays used in our study, the Slim Straight (E12: 24 mm) and the Flex24 (24 mm).

### Hearing preservation in children

Only a small number of published studies exist on pediatric patients with sufficient low-frequency residual hearing and formal indication for EAS provision using structure-preserving CI electrode arrays.

Some clinics have a cautious approach towards EAS provision as the measurement of auditory thresholds basically always entails uncertainty in early childhood. Furthermore, some clinicians are of the opinion that it is better to implant a longer electrode array a priori in cases of progressive inner ear HL. Nevertheless, in the current study the possibility of EAS provision to small children is presented, since it can be assumed that especially children may benefit from better rehabilitation perspectives in the future. As a larger number of cases is needed, joint prospective long-term observations at different clinics seem useful [18].

The study of Brown et al. showed hearing preservation in 31 children (mean age: 9.9 years) [3]. The median PTA at 250, 500, and 1000 Hz deteriorated by 20 dB from 83.3 dB to 103 dB after implantation. The audiological measurements were performed after an average of 10.3 months (range: 1–30 months). In that study, however, only conventional types of electrode arrays that are longer and bulkier were used; therefore, they are not primarily designed for hearing preservation cochlear implantation. The mean preoperative HL of 83.3 dB was also very likely beyond the classical EAS indication (auditory thresholds in quiet <500 Hz of 0–60 dB HL, [10]). This may explain the relatively large deterioration of the postoperative PTA in that study.

In a retrospective study Bruce et al. showed hearing preservation in 14 adolescents (mean age: 13.5 years), achieved through the soft surgery procedure [4]. The PTA_low_ deteriorated by 11.1 dB from 52.0 to 63.9 dB. Compared to our study, the preoperative baseline HL was clearly more distinct (13 dB higher). Because of the distinct HL, the Flex20/Flex24 electrode arrays specifically developed for EAS and HPCI was only used for implantation in 5 patients of the Bruce et al. study. The other 9 patients were implanted with the considerably longer FlexSoft (31.5 mm) or the Flex28 electrode arrays; therefore, the results of Bruce et al. cannot be directly compared to the data presented in our study.

Benghalem et al. reported on cochlear implantations in 7 children (mean age: 4.5 years) which were performed using the Mid-Scala Electrode from Advanced Bionics (Stäfa, Switzerland) designed for hearing preservation cochlear implantation according to the HPCI standard [2]. As some of the patients were very young (<4 years), it was not possible to create an authentic audiogram for both ears in this patient population; the contralateral ear was only blocked with an adapted silicone piece and thus insufficiently “deafened”. Six of the 7 children had at least partial hearing preservation with a deterioration <10 dB HL at the frequencies of 500 Hz and 1000 Hz. The hearing preservation calculated following Skarzynski [25] was only calculated for the 500 Hz frequency; it was complete in 3 patients, partial in 3 patients and minimal in 1 patient.

Carlson et al. also presented the results after cochlear implantation in 35 children (43 ears) with a mean age of 8.6 years [5]. In that study, the hearing preservation was analyzed although mostly cases with conventional “long” electrode arrays instead of EAS electrode arrays were analyzed. Furthermore, the implantation was not always performed according to the HPCI standard for other reasons: In 51.2%, for example, a cochleostomy was performed during surgery. The preoperative PTA_low_ deteriorated by 25.3 dB from 54.2 dB to 79.5 dB at frequencies of 250 Hz and 500 Hz at the postoperative measurement of auditory thresholds 10.7 months after implantation. The calculated hearing preservation of the low-frequency residual hearing following Skarzynski [25] was complete in 17 ears (39.5%) and partial in 19 ears (44.2%). Complete HL was identified in 7 ears (16.3%). Analyzing the long-term results of the audiometric thresholds postoperatively over time (mean: 43.8 months; range: 2.6–108.3 months), the PTA_low_ deteriorated by another 9.7 dB on average.

Further improvements in hearing preservation could probably be achieved by using new procedures to insert the electrode array. Stuermer et al. [26], for example, described the “underwater technique” which entails filling the tympanic cavity with Ringer’s solution to avoid hydrostatic pressure shocks of the cochlea during insertion of the electrode array. In order to ensure successful pressure balance between the fluids through the round window (during insertion of the electrode array), however, the round window must be opened widely, which may further result in deterioration of the auditory thresholds.

Intracochlear pressure waves, caused by the insertion of the CI electrode array into the cochlea, can be reduced by an appropriate design of the electrode array. Pressure measurements by Mittmann et al. [14] in an artificial cochlea model showed that pressure shocks during insertion are less intense when using electrodes placed in the middle of the scala tympani than when using electrodes placed adjacent to the lateral wall of the cochlea. This could further improve hearing preservation after cochlear implantation.

### Limitations

The main goal of the present study was to analyze the hearing preservation results in children with EAS indication provided with a cochlear implant. Speech understanding outcomes are therefore not part of the retrospective analysis. Due to the retrospective study design, the availability of postoperative audiograms is limited in some cases, and the times of analysis inevitably vary to a certain extent. This limits the conclusions that can be drawn with regard to the actual hearing preservation in the observed cohort.

## Outlook

The present study provides evidence that hearing preservation can be achieved after cochlear implantation in children. To achieve this, the implantation should be performed following the HPCI standard. Providing children with low-frequency residual hearing with electric-acoustic stimulation (EAS) should be aimed for as a high level of hearing preservation can also be achieved in children over the medium and long term.

## Practical conclusion

- Low-frequency hearing preservation can be achieved after cochlear implantation in children.
- Hearing preservation surgical techniques and hearing preservation electrode array designs must be used.
- Children should be informed about the expected success of EAS provision with regard to better speech understanding, even though speech audiometry in early childhood is not an option for early performance measurement.
