# Erratum to: Hearing preservation in children with electric-acoustic stimulation after cochlear implantation: Outcome after electrode insertion with minimal insertion trauma

**DOI:** 10.1007/s00106-020-00844-x

**Published:** 2020-03-05

**Authors:** T. Rader, A. Bohnert, C. Matthias, D. Koutsimpelas, M-A. Kainz, S. Strieth

**Affiliations:** 1 Department of Otorhinolaryngology – Section of Audiology, Ludwig-Maximilian-University Medical Center, Marchioninistr. 15, 81377 Munich, Germany; 2 Audiological Acoustics Division, Department of Otolaryngology, Head and Neck Surgery, University of Mainz, Langenbeckstraße 1, 55131 Mainz, Germany; 3 Department of Otolaryngology, Head and Neck Surgery, University of Mainz, Mainz, Germany

**Erratum to:**

**HNO 2018**

10.1007/s00106-018-0532-3

The article *Hearing preservation in children with electric-acoustic stimulation after cochlear implantation*—*Outcome after electrode insertion with minimal insertion trauma*, written by T. Rader, A. Bohnert, C. Matthias, D. Koutsimpelas, M‑A. Kainz, S. Strieth was originally published electronically on the publisher’s internet portal (currently SpringerLink) on 21 August 2018 without open access.

With the author(s)’ decision to opt for Open Choice the copyright of the article changed on February 2020 to © The Author(s) 2020 and the article is forthwith distributed under the terms of the Creative Commons Attribution 4.0 International License (http://creativecommons.org/licenses/by/4.0/), which permits use, duplication, adaptation, distribution, and reproduction in any medium or format, as long as you give appropriate credit to the original author(s) and the source, provide a link to the Creative Commons license and indicate if changes were made.

The original article has been corrected.

